# The Possible Association Between Bisphenol A (BPA) and the Neuropathological Processes Characteristic of Alzheimer’s Disease—A Systematic Review of the Literature

**DOI:** 10.3390/ijms27157026

**Published:** 2026-08-05

**Authors:** Marta Żebrowska-Gamdzyk, Mateusz Maciejczyk

**Affiliations:** 1 Faculty of Health Sciences, University of Lomza, Akademicka Street 14, 18-499 Lomza, Poland; 2 Faculty of Medical and Health Sciences, University of Siedlce, 08-110 Siedlce, Poland

**Keywords:** Alzheimer disease, bisphenol-A, BPA, neurodegenerative processes

## Abstract

Bisphenol A (BPA) is a ubiquitous environmental endocrine-disrupting compound whose potential neurotoxicity is attracting increasing interest. A growing body of evidence suggests a possible link between BPA exposure and neurodegenerative processes; however, comprehensive analyses of its role in Alzheimer’s disease (AD) are lacking. The aim of this systematic review was to evaluate the available scientific evidence regarding the association between BPA exposure and Alzheimer’s disease. A systematic literature review was conducted in accordance with the PRISMA 2020 guidelines. The PubMed/MEDLINE, Scopus, and Web of Science databases were searched up to 23 July 2026. Original research articles and meta-analyses published in English were included, covering studies in humans and animal models that analyzed the effects of BPA on Alzheimer’s disease or related mechanisms. Study selection and data extraction were performed independently by two reviewers. The risk of bias was assessed using the ROBINS-E and SYRCLE tools. Of the 70 publications identified, 12 studies were included in the analysis. Most data came from in vivo and in vitro studies, and one study was clinical in nature (autopsy-based). The findings indicate that BPA exposure is associated with cognitive dysfunction, increased oxidative stress and neuroinflammation, amyloid-β accumulation, and tau protein hyperphosphorylation. Identified mechanisms included, among others, disturbances in insulin signaling, activation of the NF-κB and STAT3 pathways, and mitochondrial dysfunction. Eighty percent of the studies were rated as having good methodological quality. The analysis was limited by study heterogeneity, the small number of clinical studies, and the inability to perform a meta-analysis. The predominance of animal models and the use of high BPA doses hinder the direct extrapolation of the results to the human population. The available evidence suggests a possible association between BPA exposure and neuropathological processes characteristic of Alzheimer’s disease. However, further well-designed epidemiological studies are needed to assess the impact of long-term, low-dose BPA exposure on the risk of developing AD.

## 1. Introduction

Alzheimer’s disease is an age-related progressive disorder characterized by cognitive impairment and memory deficits [1]. It is the leading cause of dementia among older adults [1,2]. It is estimated that approximately 39 million people worldwide are currently affected by Alzheimer’s disease [3]. Due to population aging, this number may reach as many as 98–106 million by 2050. The growing number of diagnosed cases of Alzheimer’s disease poses a major challenge to healthcare systems [3].

Two forms of Alzheimer’s disease are distinguished. The first is the familial, early-onset form (FAD, Familial Alzheimer’s Disease), which accounts for approximately 1% of all cases. This form is associated with inherited genomic alterations [4]. The second form is the spontaneous (sporadic) type—SAD (Sporadic Alzheimer’s Disease)—which accounts for as many as 99% of diagnosed cases. SAD has a later onset than FAD, and its etiology is complex [5]. Genetic predisposition plays a crucial role as a risk factor for Alzheimer’s disease, accounting for an estimated 58–79% of cases. Rare mutations in the *APP* (*β-Amyloid precursor protein*), PSEN1 (*Presenilin 1*), and PSEN2 (*Presenilin 2*) genes are associated with the autosomal dominant form of AD. In contrast, the *apolipoprotein E* (*APOE*) gene constitutes the major risk factor for the sporadic form of the disease [6]. However, age remains the most significant risk factor for the development of AD. The disease affects 18.1% of individuals over 65 years of age, with this rate rising to 33.2% among those over 85. Furthermore, it should be noted that women are more susceptible to developing the condition than men [7].

The Lancet Commission on Dementia Prevention also identified 12 modifiable factors that collectively account for approximately 40% of dementia cases worldwide. These include low education levels, hypertension, hearing impairment, tobacco abuse, obesity, depression, physical inactivity, type 2 diabetes, and limited social contact [1,2,3,4,5,7,8]. In recent years, attention has also been drawn to lifestyle-related factors, including imbalance between work and rest, stress, reduced sleep duration, substance use, limited intellectual and social activity, as well as civilizational changes and poor diet [7,8]. Studies indicate that the presence of polyphenols, vitamins, and other compounds with antioxidant properties in the diet reduces the risk of developing Alzheimer’s disease, whereas excessive intake of saturated fatty acids accelerates disease progression [6,7,8]. Environmental risk factors for Alzheimer’s disease also include cigarette smoke, environmental pollution, endocrine-disrupting chemicals, heavy metals, and pesticides [6].

**Figure 1:**
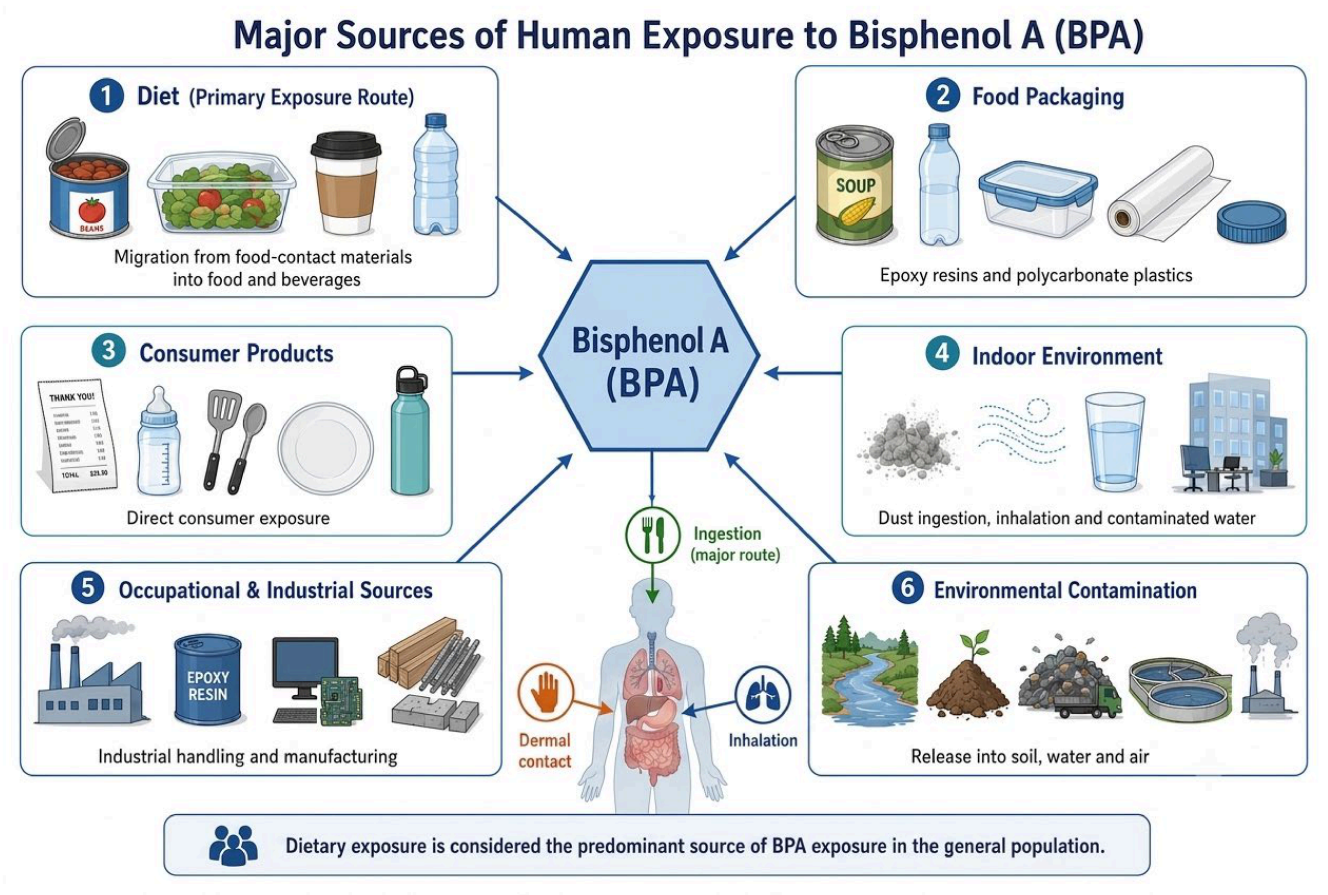
Sources of BPA.

Epidemiological studies and animal reports indicate an association between exposure to bisphenol A (BPA; 4,4′-dihydroxy-2,2-diphenylpropane) and cognitive–behavioral changes [9,10,11]. BPA is a propane derivative with widespread industrial applications. It is used, among others, in the production of thermal paper, pesticides, leather tanning agents, medical devices, and electronic products, and it is also used to coat the interior of food cans. Such extensive use has resulted in BPA being widely distributed in the environment [11]. Studies show that BPA can be detected in water, soil, and air; however, the greatest human exposure occurs through the consumption of food. Its migration from packaging can unintentionally contaminate food products, affecting the human body via inhalation, ingestion, and direct contact (Figure 1) [12]. BPA is metabolized in the liver to bisphenol A glucuronide, which is then excreted in the urine. BPA acts as an agonist or antagonist of estrogen receptors [13]. Consequently, its role has been demonstrated in both male and female infertility [11,13], as well as in precocious puberty [12,14] hormone-dependent cancers (e.g., breast and prostate cancer) [15,16], and polycystic ovary syndrome (PCOS) [16,17,18]. Measurement of BPA concentration in urine is one of the best methods for assessing exposure to this compound. Continuous daily exposure and the tendency of BPA to bioaccumulate appear to pose a particular threat to health [19].

Recent studies have shown that prenatal exposure to BPA is associated with abnormalities in the brain and nervous system of the offspring. Notably, BPA neurotoxicity is observed not only during early developmental stages but also leads to alterations in neuronal morphology in adulthood [20]. There is increasing evidence that in utero exposure to BPA has far-reaching effects that occur not only in the F1 generation but also in the F2 and F3 generations [20,21,22,23]. Moreover, a growing body of data indicates an association between environmental exposure to BPA and neurodegenerative diseases [24]. However, there is a lack of studies summarizing current knowledge on the role of BPA in individuals with Alzheimer’s disease. Given that Alzheimer’s disease represents one of the greatest public health challenges of the 21st century, it is essential to identify all factors influencing its occurrence. Therefore, the aim of this study is to conduct a systematic review of the literature on the relationship between BPA exposure and neuropathological processes characteristic of Alzheimer’s disease.

## 2. Methods

The literature search was conducted between 25 June and 1 August 2026. It was performed in accordance with the Preferred Reporting Items for Systematic Reviews and Meta-Analyses (PRISMA 2020) guidelines [25], using the PubMed/MEDLINE, Scopus, and Web of Science databases. Only publications in English were evaluated.

The available literature was reviewed using the following keywords: “BPA” and “Alzheimer’s disease,” “bisphenol A” and “Alzheimer’s disease,” “BPA” and “dementia,” “BPA” and “neurodegenerative diseases,” and “BPA” and “neurological system.” Studies included in the review comprised original research articles and meta-analyses published in English evaluating the link between BPA exposure at various stages of life (prenatal, childhood, and adulthood) and the risk of Alzheimer’s disease in human and animal models. Exclusion criteria comprised: articles published in languages other than English, review articles, and studies investigating the effects of BPA exposure on diseases other than Alzheimer’s disease.

This systematic review was conducted and reported in accordance with the Preferred Reporting Items for Systematic Reviews and Meta-Analyses (PRISMA) 2020 guidelines. The protocol of this systematic review was not prospectively registered in a public review registry.

### 2.1. Data Extraction

The initial data screening was performed independently by two authors based on titles and abstracts. Subsequently, the full texts of the selected publications were reviewed. All studies that met the eligibility criteria were included in the present review. In cases of uncertainty, the authors consulted with each other. To ensure data quality, all publications were assessed for methodological rigor, and the following variables were recorded: authors, year of publication, study design, and study outcomes.

**Figure 2:**
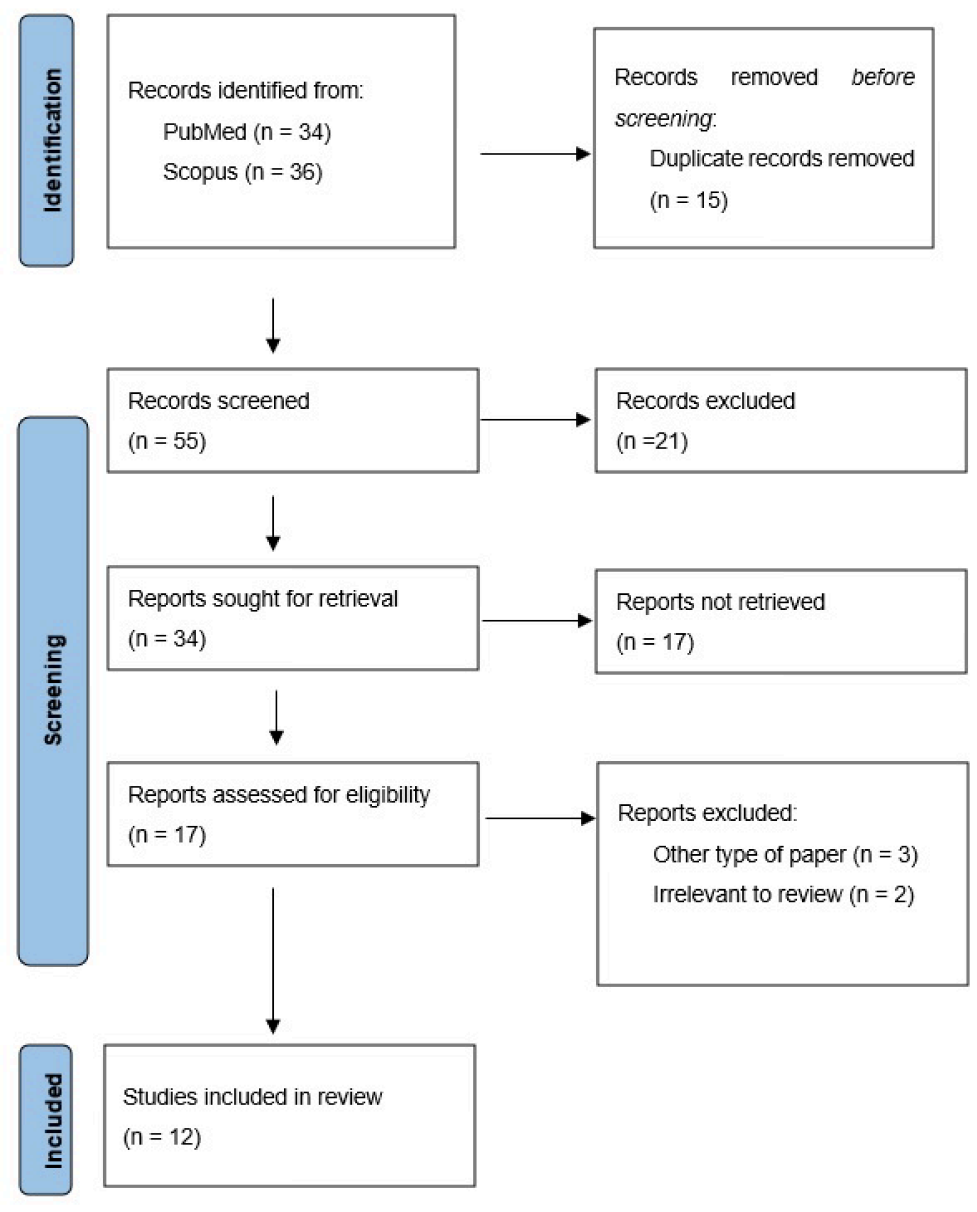
PRISMA (Preferred Reporting Items for Systematic Reviews and Meta-Analyses) flow diagram presenting the search strategy [25].

The literature search identified 70 articles from the PubMed and Scopus databases, of which 15 were removed as duplicates. A total of 55 abstracts were screened, and 34 met the inclusion criteria. Among the eligible studies, 5 were deemed irrelevant to this review or represented publication types other than those sought. This resulted in 12 studies being included in the review. The PRISMA flow diagram illustrating the study selection strategy is presented in Figure 2.

### 2.2. Risk of Bias Assessment

**Figure 3:**
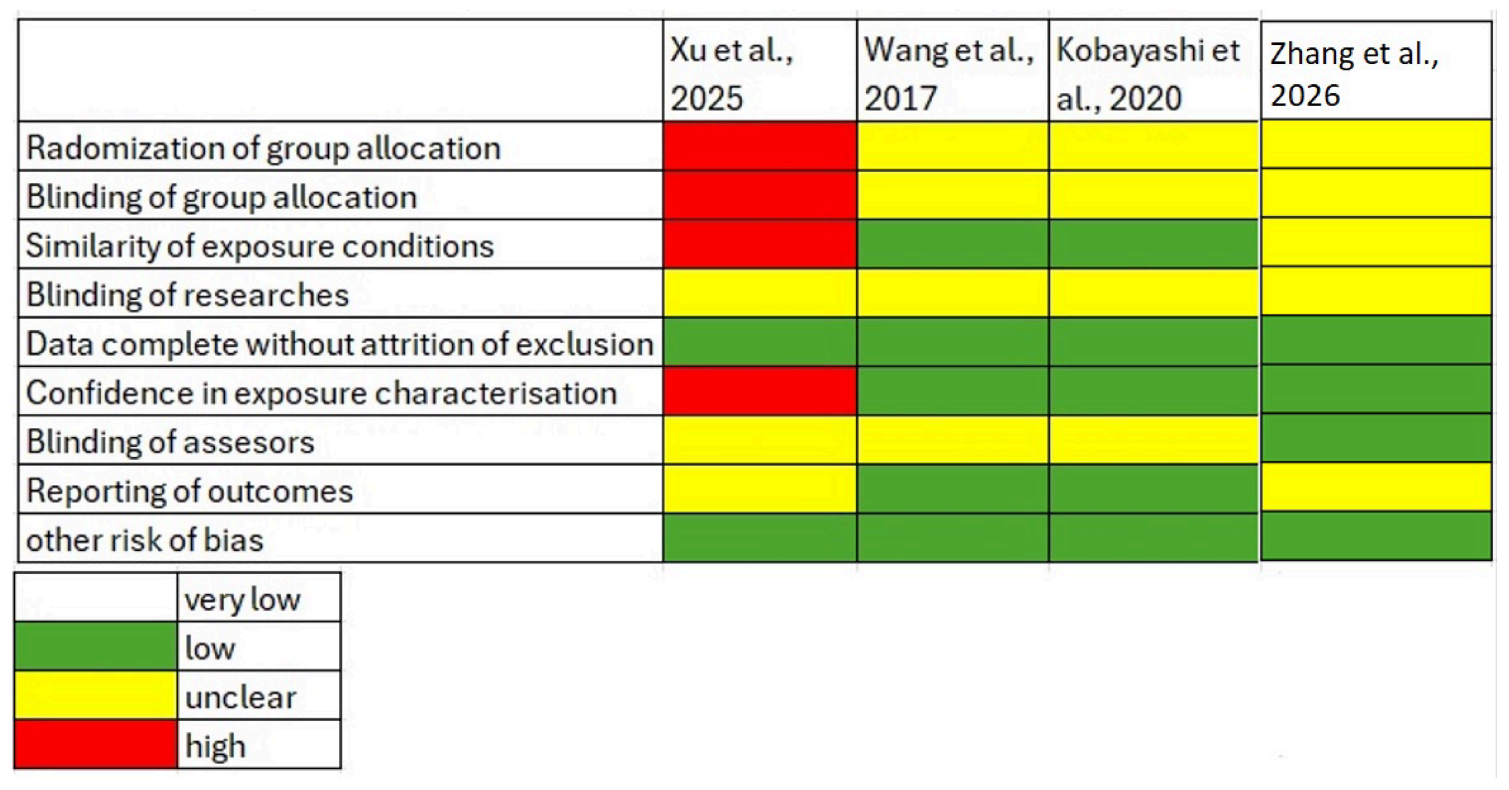
Risk of bias assessment of the in vitro and in silico studies [28,29,30,31].

**Figure 4:**
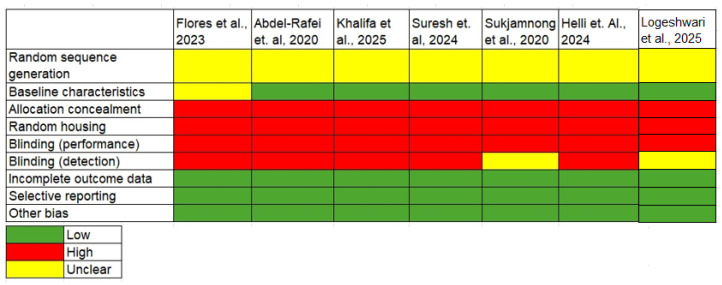
Risk of bias assessment of the animal studies [32,33,34,35,36,37,38].

**Figure 5:**
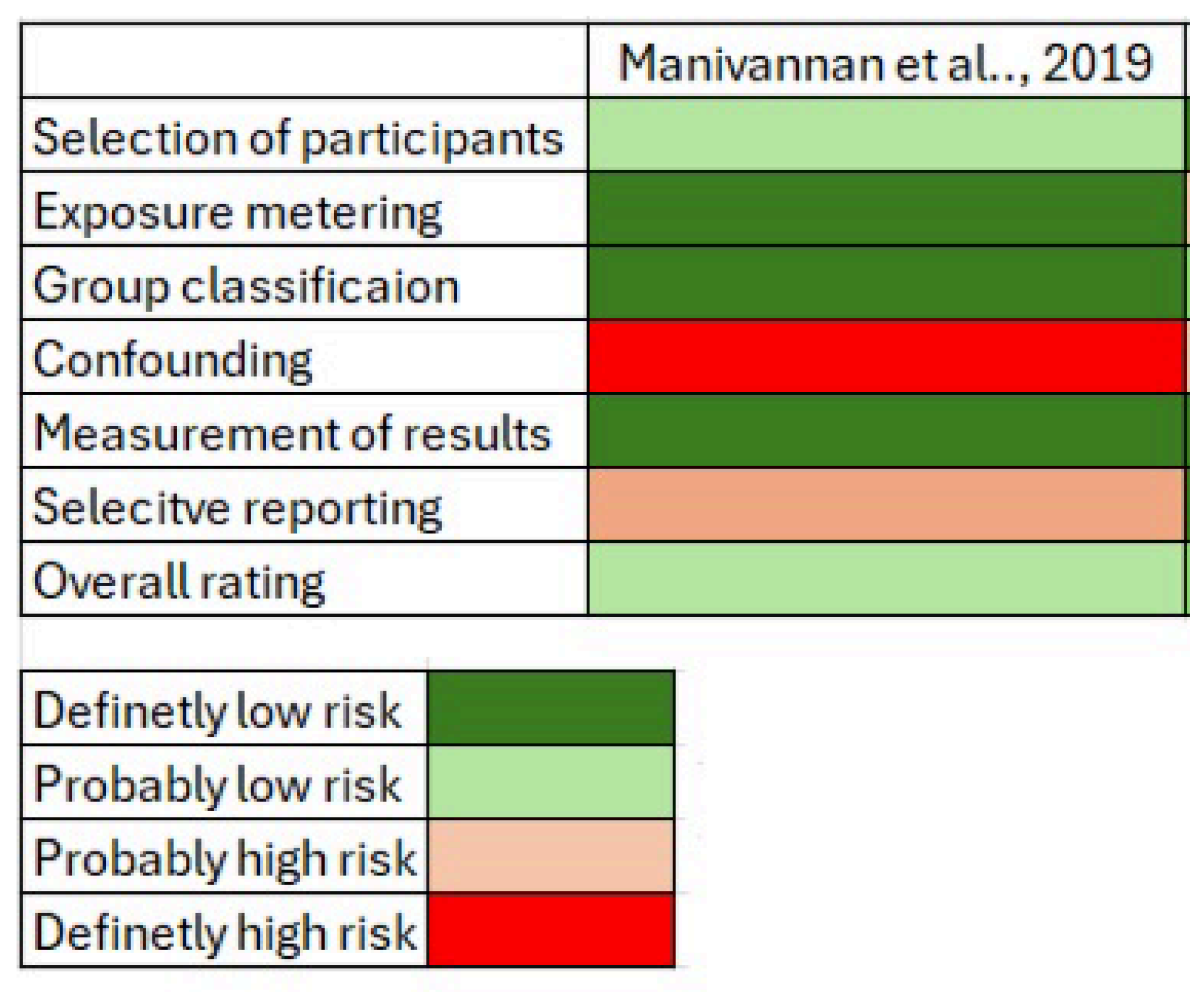
Risk of bias assessment of the postmortem human studies [39].

To assess the risk of bias in each study, an analysis using the Risk Of Bias In Non-randomized Studies of Exposure (ROBINS-E) tool [23], SYRCLE [26] (SYstematic Review Center for Laboratory Animal Experimentation) for animal studies, and a third analysis for in vitro studies were performed [27]. The questionnaires were completed by two independent reviewers, and any discrepancies were resolved through discussion. A summary of the risk-of-bias assessment for each study is presented in Figure 3, Figure 4 and Figure 5.

The most frequently identified sources of bias were the lack of justification for sample size and the absence of a control group. The overall critical appraisal was summarized by summing the scores for each potential risk criterion (scores: 1—low risk, 0.5—unclear risk, 0—high risk). The majority of the studies (80%) were rated as having good methodological quality.

Risk of bias assessment showed that both the bioinformatics study by Xu et al. [28] and the in vitro experiment by Wang et al. [30] were characterized by a moderate risk of bias. The work by Xu et al. provides predictive information about potential pathways and molecular targets of BPA, but the lack of experimental validation is a significant limitation. In contrast, the study by Wang et al. presents robust in vitro mechanistic data. The study by Kobayashi et al. [31] presents good cellular data.

Risk of bias assessment (Figure 4) revealed that all included studies had a moderate or moderately high risk of bias. The most frequently identified limitations were problems with allocation concealment, random housing or blinding.

The study by Manivannan et al. [39] was assessed as having a moderate risk of bias (Figure 5). The measurement of persistent organic pollutant exposure in postmortem tissues was objective and precise, but important confounding factors were not controlled for.

### 2.3. Limitations of the Studies and the Systematic Review

Unfortunately, due to the substantial heterogeneity of the studies, it was not possible to apply advanced statistical methods to the data analysis that could have improved the quality of the review and the strength of the conclusions (i.e., meta-analysis). A major limitation of the analyzed literature is the small number of studies conducted in human models, including the lack of randomized controlled trials and cohort studies. Data derived from autopsy studies are cross-sectional in nature and do not allow for the establishment of a causal relationship between BPA exposure and the development of Alzheimer’s disease. It should also be noted that numerous confounding factors (such as age, diet, environmental pollution, or coexisting diseases) may have influenced the reported outcomes. It should also be noted that exposure to BPA undoubtedly causes disorders similar to those occurring in Alzheimer’s disease, but as far as the disease itself is concerned, there is still no clear evidence.

The vast majority of the analyzed data originate from in vitro or in vivo studies using animal models (primarily rats), which limits the direct extrapolation of the findings to the human population. More specifically, the studies employed varying BPA doses and exposure durations, making objective comparison of results difficult. Some experiments used short-term, acute BPA exposure, which does not reflect the chronic, low-dose exposure most commonly observed in human populations. It should be remembered that the doses used in animal studies (0.04 mg/kg–150 mg/kg) are much higher than typical human exposure (tolerable daily intake for humans for BPA is 0.0000002 mg/kg [40]), therefore the conclusions drawn from these studies cannot be clearly applied to the human model. The routes of administration also differed from typical human exposure to BPA, which may affect its pharmacokinetics, metabolism, and concentrations in target tissues. It should also be emphasized that many of the analyzed studies relied solely on indirect markers (such as biomarkers of oxidative stress, inflammation, or neuropathogenic proteins). Although these biomarkers indicate neurotoxicity, they do not unequivocally demonstrate the presence of full-blown Alzheimer’s disease.

### 2.4. Next Steps and Future Directions

In the European Union and the United States, the use of BPA has been banned in infant feeding bottles and in food packaging intended for infants [41,42]. The United States Environmental Protection Agency (EPA) has established a reference dose for BPA of 50 μg/kg body weight/day [42]. However, due to the limited number of studies in clinical models, it remains unclear whether such BPA doses influence the development of Alzheimer’s disease. It is therefore necessary to determine exposure levels that do not induce neurotoxic changes. Although an association between BPA and neurodegeneration has been suggested, there is a lack of large, prospective epidemiological studies that account for multiple confounding factors such as age, sex, and coexisting metabolic diseases. Moreover, it is unknown whether neurodegenerative changes in Alzheimer’s disease are a consequence of metabolic disturbances induced by BPA exposure or the direct effects of BPA on brain cells.

Although the available literature indicates that BPA affects numerous neuropathogenic signaling pathways (e.g., NF-κB, STAT3, PGC-1α/AMPK), the precise mechanisms of BPA action in brain cells remain unclear. Further research is required in this area, using modern approaches such as bioinformatics analyses and omics-based studies.

It is particularly important to gain a better understanding of the effects of long-term exposure of human populations to low doses of bisphenol A. Currently, much of the available data come from preclinical studies, limiting the ability to fully extrapolate results to clinical and population-based settings. Experimental studies use very high doses of BPA. Therefore, there is a clear need for well-designed epidemiological studies and prospective cohort studies in humans to assess the long-term health effects of such exposure. It is particularly important to consider chronic and low-dose exposure, which can reflect real-world environmental conditions. Future research should also focus on identifying potential high-risk groups, such as children, pregnant women, and those exposed occupationally. Integrating experimental findings with observational data from population studies could significantly contribute to a better understanding of the mechanisms of action of BPA and its long-term health consequences.

Currently, BPA is being gradually replaced with potentially safer analogs such as bisphenol S (BPS), bisphenol F (BPF), and bisphenol AF (BPAF) [6,43]. However, the effects of these derivatives on the development of neurodegenerative diseases, including Alzheimer’s disease, are unknown. Before their widespread use, it is essential to thoroughly investigate whether their impact on the human body is similar to that of BPA. If comparable toxicity is identified, continued efforts to identify new alternative materials will be necessary.

The development of therapeutic strategies to mitigate the neurotoxic effects of BPA on the human body is also needed.

## 3. Results and Discussion

Since the 1960s, BPA has been used to coat the interiors of plastics and cans, making it one of the most widely used chemicals by humans [24]. Bisphenols also enter the body through contaminated food or water, and contact with thermal paper receipts is another significant source of exposure. The effects of BPA on the reproductive system, infant development, and metabolic disorders—including obesity, endocrine disruption, and impacts on the nervous system—have been described [23,44,45]. However, relatively little is known about the relationship between BPA exposure and the incidence of Alzheimer’s disease.

The aim of this systematic review was to evaluate the available scientific evidence regarding the association between BPA exposure and neuropathological processes characteristic of Alzheimer’s disease. Twelve studies were ultimately included in the review, including two in vitro and in silico studies, seven animal studies, and one postmortem study. A description of the studies is provided in Table 1.

Analysis of available data suggests that BPA may contribute to the development of AD-like pathological changes through multiple interconnected mechanisms rather than through a single molecular pathway. The most consistently observed mechanism in experimental studies is the induction of oxidative stress, which may represent an initial trigger for subsequent pathological processes, including neuroinflammation, mitochondrial dysfunction, impaired glucose metabolism, and accumulation of Aβ and hyperphosphorylated tau [12]. Therefore, BPA may act as a factor that enhances existing neuronal vulnerability to neurodegenerative processes rather than as an independent initiator of disease.

In a study by Flores et al. [32], Wistar rats were administered BPA at a dose of 40 μg/kg. Fourteen days of exposure to the toxin resulted in loss of cholinergic neurons in the forebrain, which regulate memory and learning processes. Their selective loss, observed in Alzheimer’s disease and other neurodegenerative disorders, leads to cognitive impairment [33,34]. A study by Kobayashi et al. [31] indicates that BPA induces S-nitrosylation of protein disulfide isomerase (PDI). Increased S-nitrosylation is a consequence of excessive production of nitric oxide (NO) due to activation of nitric oxide synthase (NOS) by BPA. It is well known that PDI S-nitrosylation is increased in the brains of patients with Alzheimer’s and Parkinson’s diseases, which directly contributes to neurodegeneration [46,47]. Interestingly, despite the substantial differences in administered doses, neurodegeneration was observed in both studies. This suggests that both low-dose chronic exposure and higher-dose short-term exposure may not be biologically neutral and could potentially contribute to adverse neurological effects in humans.

Logeshwari et al. [38] demonstrated that BPA exposure induced significant behavioral impairments in the novel tank diving test, as evidenced by reduced total distance traveled, fewer line crossings, and prolonged latency before entering the upper zone. These behavioral alterations were successfully reversed following supplementation with karanjin (furanoflavonoid isolated from *Pongamia pinnata*). At the biochemical level, BPA-exposed groups exhibited marked dysregulation of the cholinergic system, reflected by a significant increase in acetylcholinesterase (AChE) activity. Furthermore, BPA toxicity induced pronounced oxidative stress and impaired antioxidant defense mechanisms, characterized by reduced total protein levels, decreased superoxide dismutase (SOD) activity, increased lipid peroxidation (LPO), and reduced glutathione (GSH) levels compared with control animals. Notably, concurrent administration of karanjin effectively attenuated BPA-induced neurotoxic and oxidative damage.

In a study conducted by Abdel-Rafei et al. [33], female rats were divided into five groups: one control group and four experimental subjected to subacute BPA intoxication or exposed to fractionated weekly doses of γ-radiation. Some groups were simultaneously treated with methylsulfonylmethane (MSM). The study suggests that combined exposure to bisphenol A (150 mg/kg/day for 4 weeks) and γ-radiation (3 Gy/week) induces changes typical of neurodegenerative disorders, namely increased oxidative stress, extensive neuroinflammation, and elevated markers of Alzheimer’s disease (amyloid beta [Aβ] and acetylcholinesterase). Histopathological examination of the cortex and hippocampus also suggests neurodegenerative changes. Treatment with MSM (400 mg/kg/day) was shown to reduce oxidative stress levels (MDA and NOx) and markers of neuroinflammation (interleukin-1β and TNFα) [33]. In a study by Hatipoglu et al. [48], rats administered BPA at a dose of 100 mg/kg body weight by gavage for 30 days also showed increased oxidative stress. GSH levels decreased in the brain and sciatic nerve tissues of the animals, while MDA levels increased. However, concomitant administration of Nigella Sativa Seed Oil increased tissue GSH content and significantly reduced MDA levels. This is also confirmed by experimental studies indicating the neuroprotective role of antioxidant-based interventions in limiting BPA-induced oxidative damage [49,50,51].

Similarly, a study by Helli et al. [37] demonstrated that exposure of rats to BPA for 21 days at doses of 50, 100, and 200 mg/kg/day altered neurobehavioral responses, increased MDA levels and oxidative stress, and reduced the activity of brain antioxidant enzymes. Western blot analysis further indicated increased expression of phosphorylated tau (p-tau) and Aβ. BPA was shown to potentially contribute to the development of Alzheimer’s disease through effects on the mitochondria-dependent apoptotic pathway, as well as the AMPK/PGC-1α/FNDC5 signaling pathway (AMPK—AMP-activated protein kinase; PGC-1α—peroxisome proliferator-activated receptor gamma coactivator 1 alpha; FNDC5—fibronectin type III domain-containing protein 5) and the CREB/BDNF/TrkB pathway (CREB—cAMP response element-binding protein; BDNF—brain-derived neurotrophic factor; TrkB—tropomyosin receptor kinase B). PGC-1α is known to regulate the expression of genes involved in reactive oxygen species production, and its phosphorylation can be regulated by AMPK; thus, this pathway may represent an interesting target for AD intervention and treatment. The CREB/BDNF pathway, in turn, plays an important role in nervous system development [52,53]. Notably, syringic acid (SA) administered at a dose of 50 mg/kg reversed the behavioral, biochemical, and molecular changes induced by high doses of BPA.

In the study by Khalifa et al. [35], Wistar rats were divided into four groups: control, BPA, BPA + ferulic acid (FA), and FA. The rats received BPA at a dose of 50 mg/kg body weight dissolved in corn oil for 40 days. The aim of the study was to evaluate the neuroprotective effect of FA against changes induced by bisphenol A exposure. The results indicated significant cognitive impairment in the BPA-exposed group. These rats also showed increased levels of brain oxidative stress biomarkers, including MDA (a marker of lipid peroxidation), GSSG (oxidized glutathione), and GSH (reduced glutathione). Histopathological examination of the brains revealed elevated tau protein levels in tissues from rats treated exclusively with BPA. Tau is a key component of neurofibrillary tangles, which are a hallmark of Alzheimer’s disease. Aggregation of phosphorylated tau (p-tau) can lead to neuronal dysfunction or death.

Similar results were obtained in studies by Suresh [36] in which cyanidin was used as a synthase kinase-3β (GSK3β) inhibitor and restored Wnt3 and β-catenin levels. In addition, cyanidin exhibited antioxidant properties by increasing catalase and superoxide dismutase activity. Cyanidin intervention significantly reduced acetylcholinesterase levels in the cerebral cortex and hippocampus compared with BPA-treated groups.

Despite differences in BPA doses applied across studies—ranging from relatively low experimental exposure in the study by Flores et al. to high toxicological doses used by Hatipoglu et al. and Helli et al.—the reported findings demonstrate a consistent pattern of biological alterations. BPA appears to disrupt redox homeostasis by increasing the generation of reactive oxygen species (ROS) and reactive nitrogen species (RNS), weakening cellular antioxidant defense mechanisms, and triggering secondary processes such as neuroinflammation, mitochondrial dysfunction, impaired cholinergic neurotransmission, and accumulation of pathological proteins associated with AD. At the same time, differences in dose and exposure duration indicate that BPA-induced neurotoxicity may depend not only on concentration but also on exposure duration, age-related susceptibility, and additional factors influencing neuronal vulnerability.

Recent studies suggest that exposure to bisphenol A may be a risk factor for insulin resistance and other metabolic disorders. Perinatal exposure to BPA at a dose of 100 μg/kg/day has been shown to contribute to peripheral insulin resistance in offspring, with brain insulin resistance becoming evident after 8 months [54]. Similarly, in adult males receiving subcutaneous BPA at a dose of 100 μg/kg body weight/day, a reduction in insulin sensitivity and decreased levels of glucose transporter proteins GLUT-1 and GLUT-3 in the brain were observed [55]. The relationship between impaired insulin signaling and Alzheimer’s disease is noteworthy. Individuals with type 2 diabetes have up to a twofold higher risk of developing Alzheimer’s disease, and impaired glucose metabolism is a common feature of the disease [56]. Type 2 diabetes is characterized by hyperglycemia, hyperinsulinemia (at least in the early stages of the disease), and insulin resistance, and is associated with cognitive decline and cerebral hypometabolism [57,58,59]. Cognitive deficits in carbohydrate metabolism disorders are thought to result from alterations in brain insulin metabolism. Type 2 diabetes may also lead to small-vessel disease, which in turn can cause dementia [60]. Protein glycation in type 2 diabetes may further enhance the accumulation of neurotoxic proteins (amyloid beta [Aβ] and p-tau) involved in Alzheimer’s disease [61]. Another publication [62] demonstrated that the increase in Alzheimer’s disease incidence may be a complication of type 2 diabetes. Estrogen receptors act as modulators of glucose homeostasis through their influence on pancreatic β-cell function [60]. A reduction in estrogen receptor levels in the brain is associated with cognitive deficits, accelerating aging processes and increasing susceptibility to Alzheimer’s disease, particularly among women [62]. Both mechanisms may be linked to reduced estradiol levels in the brain. Environmental pollutants, including bisphenol A, have been shown to exert pharmacological effects similar to estradiol, potentially leading to increased insulin biosynthesis. BPA-induced hyperinsulinemia promotes the translocation of glucose transporter 4 (GLUT4), which is located in intracellular vesicles alongside insulin-regulated aminopeptidase (IRAP). IRAP is a receptor for angiotensin IV, which links the brain renin–angiotensin system to Alzheimer’s disease, as it is involved in emotional responses, learning processes, memory, and sensory information processing [63]. Thus, IRAP/GLUT4 may represent a potential target for pharmacological interventions against Alzheimer’s disease [62].

The study by Zhang et al. [29] provides new insights into the shared and distinct mechanisms underlying the neurotoxic effects of BPA and DEHP in Alzheimer’s disease. The identified common molecular targets and signaling pathways provide a foundation for further research into the mechanisms involved in the development and progression of Alzheimer’s disease. Wang et al. [30] demonstrated in an in vivo animal model that BPA exposure was associated with increased expression of APP, Aβ1–42, BACE-1, and phosphorylated tau proteins, accompanied by disturbances in insulin signaling pathways. Complementary in vitro experiments supported the involvement of molecular mechanisms related to oxidative stress and dysregulated insulin signaling in BPA-induced neurotoxicity [30,51].

These mechanisms are supported by the study of Sumei et al. [28], which aimed to identify potential toxicological targets and molecular mechanisms of BPA-induced Alzheimer’s disease. Using systematic bioinformatics analyses, 137 potential targets for BPA-induced Alzheimer’s disease were identified. Among the most important were STAT3 (signal transducer and activator of transcription), AKT1, insulin, EGFR, and PTEN. STAT proteins are cytoplasmic transcription factors that regulate a wide range of genes. STAT3 is particularly important, as it influences the biological functions of neurons and the hippocampus—key cell types involved in AD progression. It also participates in modulating levels of molecules such as Aβ42 and BACE1, which are associated with Alzheimer’s disease. Clinically, patients with Alzheimer’s disease show reduced expression of phosphorylated STAT3, which plays a crucial role in disease progression [60].

Considering the high prevalence of type 2 diabetes mellitus in the general population and the increasing contribution of lifestyle-related risk factors, this pathway may represent an important mechanism requiring further investigation. Both population aging and the rising prevalence of overweight, obesity, and metabolic disturbances are concerning, as these factors represent major risk factors for AD development, and additional exposure to BPA may further increase this vulnerability [12,64].

The above data should be interpreted with caution, as most of the studies were conducted in an animal model using much higher doses of BPA than typical environmental exposures in humans. According to EFSA data, the highest BPA exposure occurs in adolescents (0.001449 mg/kg/day), infants and young children (0.000875 mg/kg/day), and women and men of reproductive age (0.000388 mg/kg/day) [65]. These are much lower doses than those used in experimental models. In a rat or mouse model, it is also difficult to observe all the symptoms of Alzheimer’s disease.

A study conducted by Sukjamnong et al. [34] investigated the effects of prenatal BPA exposure on Alzheimer’s disease-related molecular mechanisms in the brains of rat offspring. Maternal exposure was applied at the NOAEL (no-observed-adverse-effect level) dose of 50 μg/kg body weight/day. A positive association was demonstrated between prenatal BPA exposure and the expression of proinflammatory genes in the hippocampus of the offspring. Maternal exposure activated the NF-κB (nuclear factor kappa-light-chain-enhancer of activated B cells) signaling pathway, which may induce neuroinflammation in the hippocampus and increase the offspring’s risk of developing Alzheimer’s disease.

Manivannan et al. [39] used tandem mass spectrometry coupled with liquid and gas chromatography to monitor concentrations of 11 environmental pollutants, including BPA, in post-mortem liver and adipose tissue samples from patients with Alzheimer’s disease. Patients were grouped according to age, and bisphenol A was one of seven toxic substances detected in 100% of the individuals studied. This study shows that environmental pollutants can be detected in AD patients, however, does not provide evidence of a causal relationship between BPA and Alzheimer’s disease. The detection of BPA in tissues of patients with Alzheimer’s disease suggests the possibility of its accumulation in the body; however, this study cannot determine whether BPA represents a cause, a consequence, or merely a marker of environmental exposure. Unlike animal models, population-based studies require consideration of numerous confounding factors, including age, diet, obesity, type 2 diabetes, and lifestyle.

**Figure 6:**
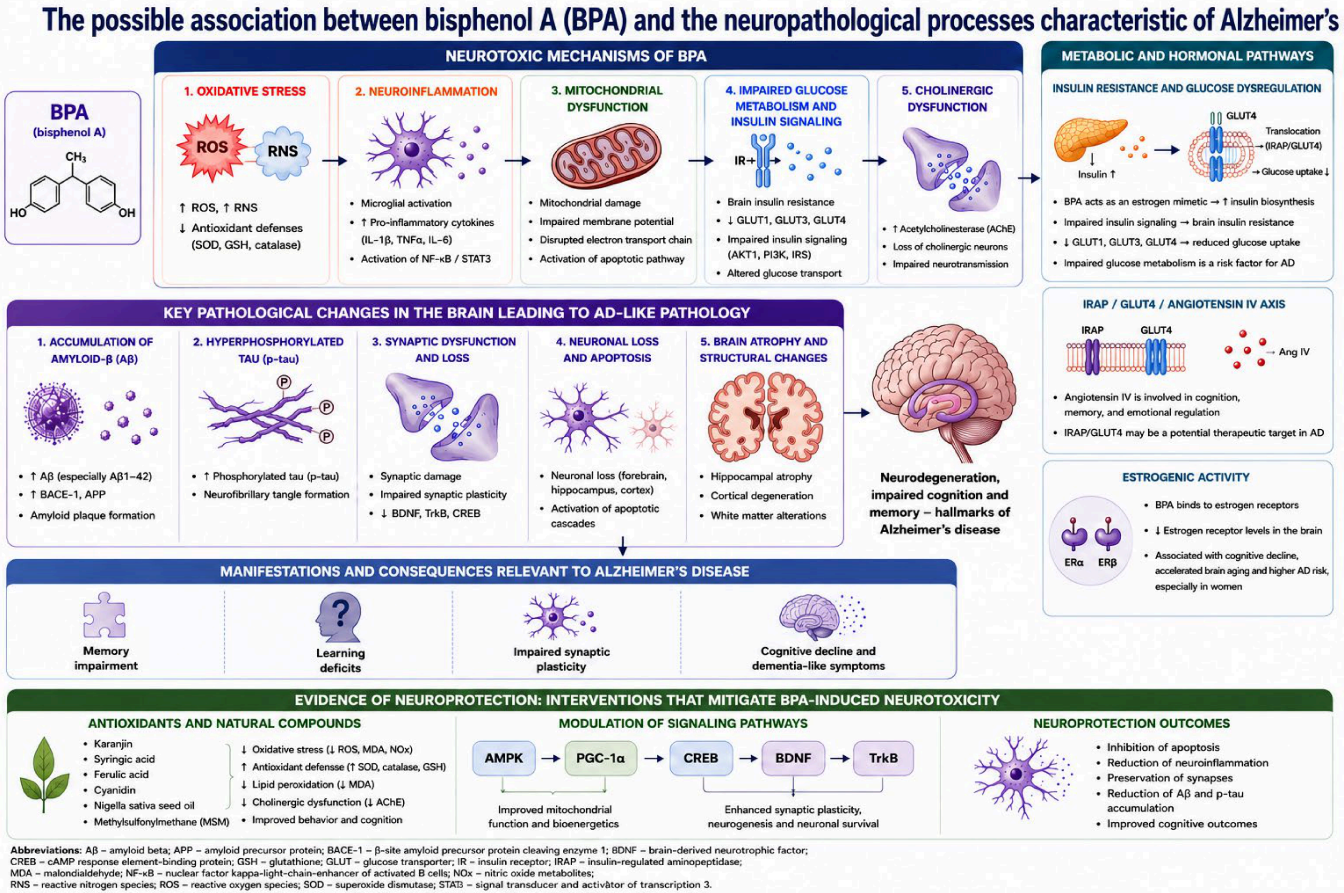
The possible association between bisphenol A (BPA) and the neuropathological processes characteristic of Alzheimer’s disease.

Although experimental studies provide strong evidence supporting the biological plausibility of BPA-induced neurotoxicity and demonstrate its ability to activate pathways associated with Alzheimer’s disease pathology, the direct translation of these findings to human disease remains challenging. Differences in BPA metabolism, exposure patterns, genetic susceptibility, and the complexity of human AD pathology must be considered. Future epidemiological and longitudinal studies are required to determine whether chronic environmental BPA exposure contributes to increased Alzheimer’s disease risk in humans (Figure 6).

## 4. Conclusions

The growing number of scientific reports on the harmful effects of BPA on human health has prompted many countries to take regulatory actions to limit the use of this compound, particularly as a component of food packaging. Although the adverse effects of BPA on the endocrine and reproductive systems, as well as its association with type 2 diabetes, have been demonstrated, it is still widely used in industry. Unfortunately, within the European Union, a complete ban on BPA applies only to infant feeding bottles.

The present review suggests a possible association between BPA exposure and the occurrence of Alzheimer’s disease-like neuropathological changes. However, the precise mechanisms by which BPA affects brain cells remain unknown. There is also a lack of large, prospective epidemiological studies assessing long-term, low-dose exposure to BPA (Figure 6).

## Figures and Tables

**Table 1:** Description of the studies included in the review.

References	Study Design	Results	Conclusions
In vitro and in silico studies			
[28]	Integrative Network Toxicology and Molecular Docking Analysis of Bisphenol A-Associated Alzheimer’s Disease	The involvement of BPA in oxidative stress induction, BPA-induced neuronal apoptosis, its contribution to neurodegenerative processes, and the association between BPA exposure and STAT3 signaling as well as insulin-related pathways highlight the potential role of BPA in the development of Alzheimer’s disease-like pathology.	Application of computational methods highlighting the complex interactions between BPA exposure and Alzheimer’s disease. The need for further experimental validation of the identified molecular targets and pathways.
[29]	A network-based toxicology approach integrating multiple databases. In vitro experimental validation of identified targets at the transcriptional and protein levels. KEGG (Kyoto Encyclopedia of Genes and Genomes) pathway enrichment analysis and molecular docking simulations	Five common key targets were identified and experimentally verified (Matrix Metalloproteinase-9 (*MMP9*), Peroxisome Proliferator-Activated Receptor Gamma (*PPARG*), Mitogen-Activated Protein Kinase 14 (*MAPK14*), B-cell lymphoma 2 *(BCL2*), (PPARG, MAPK14, BCL2, BCL2-like protein (*BCL2*)1). Both BPA and DEHP modulated these targets, influencing gene and protein expression. A common involvement in the lipid and atherosclerotic pathways was observed, as well as a compound-specific role in PI3K-Akt and MAPK signaling.	This study provides new mechanistic insights into the overlapping and distinct neurotoxic effects of BPA and DEHP in Alzheimer’s disease. The identified common targets and pathways provide a basis for further research into the mechanisms of Alzheimer’s disease.
[30]	SH-SY5Y cells were incubated with bisphenol A (BPA) at concentrations of 0, 2, 20, 200, or 2000 nM for 12 h. Phosphorylation of the insulin receptor (IR) at Tyr1355 (p-IR Tyr1355) was then detected by Western blotting. BPA-induced disturbances in insulin signaling were further evaluated at different time points (0, 1, 3, 6, 12, and 24 h).	BPA impaired insulin pathways, transiently elevating pY896-IRS1 while decreasing p-AKT (Ser473) from 6 h. Ca^2+^ influx and ROS production increased, leading to depleted ATP and reduced membrane potential. BPA dose- and time-dependently up-regulated APP expression and tau phosphorylation (pT205-tau), both peaking at 20 nM and 12 h.	This study elucidates a mechanism of pathological protein expression that may contribute to the development of Alzheimer’s disease-like pathology. These findings may provide a basis for the development of novel therapeutic strategies.
[31]	Rats were administered BPA at a dose of 100 mg/kg body weight for 6 days. Hippocampal brain homogenates were subsequently analyzed.	BPA induced S-nitrosylation of PDI in the brains of the animals.	PDI levels are elevated in patients with Alzheimer’s disease. BPA administration increased PDI levels, suggesting that excessive BPA exposure may elevate the risk of developing Alzheimer’s disease.
Animal studies			
[32]	Sixty-day-old Wistar rats were used in this study and received a single dose of BPA at 40 µg/kg. After 48 h, the basal forebrain regions were histologically examined, and the numbers of cholinergic neurons and total neurons were analyzed.	BPA caused cholinergic neuron loss, SN56 cell death, and synaptic impairment (decreased PSD-95, synaptophysin, spinophilin, NMDAR1). This was coupled with altered glutamatergic signaling (increased glutamate/glutaminase, decreased VGLUT2) and suppressed WNT/β-catenin pathways.	Further studies are required. These findings may help elucidate BPA plasticizer-induced synaptic plasticity alterations, cognitive dysfunction, and neurodegeneration, thereby contributing to their prevention.
[33]	Female rats were assigned to a control group, while the remaining four groups were subjected to subacute BPA intoxication (150 mg/kg/day) and/or exposed to fractionated weekly doses of radiation (3 Gy/week) for 4 weeks, and were either untreated or concurrently treated with MSM (400 mg/day).	Following exposure to BPA and radiation, increased oxidative stress, extensive neuroinflammation, and elevated Alzheimer’s disease markers (β-amyloid) were observed. Histopathological alterations in the cerebral cortex and hippocampus were also identified, along with pronounced Aβ deposition in the hippocampus. In contrast, MSM treatment ameliorated histopathological abnormalities and improved levels of oxidative stress, neuroinflammation, and Alzheimer’s disease markers.	These findings suggest that MSM exerts neuroprotective effects in the context of BPA and radiation exposure.
[34]	Offspring of Wistar rats prenatally exposed to BPA (5000 μg/kg) underwent hippocampal RNA-seq profiling to identify DEGs, which were then mapped against key AD-associated genes from AlzBase.	A link was demonstrated between prenatal BPA exposure and hippocampal gene expression profiles in the offspring. Maternal exposure led to increased levels of NF-κB protein, which may trigger neuroinflammation in the hippocampus and elevate the offspring’s risk of developing Alzheimer’s disease.	Prenatal BPA exposure dysregulates genes linked to AD neuropathology and neuroinflammation in offspring, suggesting potential environmental links to both AD and autism, with sex-specific effects requiring further study.
[35]	Rats were administered BPA at a dose of 50 mg/kg body weight for 40 days.	BPA exposure was associated with impaired cognitive function, increased markers of oxidative stress in the brain, elevated β-amyloid levels, increased TNF-α expression, and higher expression of total tau (tTau), indicating the involvement of BPA in processes related to neurodegeneration and Alzheimer’s disease-like pathology.	BPA impairs brain function. FA demonstrates the ability to alleviate cognitive deficits and other impairments induced by BPA.
[36]	Rats were administered BPA at a dose of 40 μg/kg, along with cyanidin at doses of 5 mg/kg or 10 mg/kg.	BPA administration resulted in memory impairment, whereas cyanidin intervention regulated phosphorylated tau (pTau) protein levels and significantly reduced acetylcholinesterase levels in the cerebral cortex and hippocampus compared with BPA-treated groups.	Exposure to BPA leads to cognitive dysfunction.
[37]	Rats were administered BPA at doses of 50 mg/kg, 100 mg/kg, or 200 mg/kg for 21 days, along with SA at a dose of 50 mg/kg.	BPA affects the mitochondria-dependent apoptotic pathway, as well as the AMPK/PGC-1α/FNDC5 and CREB/BDNF/TrkB signaling pathways and increases the expression of tau and Aβ proteins.	BPA may contribute to the development of Alzheimer’s disease, whereas SA may reduce BPA-induced toxicity.
[38]	Adult zebrafish were exposed to 4 mg/L BPA for 21 days, followed by karanjin supplementation at 5 mg/L and 10 mg/L.	BPA reduced mobility and exploration in the novel tank test while increasing AChE activity, indicating cholinergic dysregulation. BPA significantly increased lipid peroxidation while decreasing SOD, GSH, and total protein levels. Karanjin supplementation effectively reversed BPA-induced behavioral abnormalities and attenuated biochemical toxicity.	BPA exposure caused significant impairments in exploratory behavior, learning, and memory, which were reversed by high-dose karanjin.
Post-mortem study			
[39]	A mass spectrometry study coupled with liquid and gas chromatography was performed on adipose tissue and liver samples from deceased patients with Alzheimer’s disease, as well as from an age-matched control group.	Bisphenol A (BPA) was present in all (100%) of the Alzheimer’s disease patients analyzed.	There is a suspicion that BPA may be a risk factor for Alzheimer’s disease.

## Data Availability

No new data were created or analyzed in this study. Data sharing is not applicable to this article.
